# Habitat-specific alternative splicing shifts and the 5mC methylation landscape of differentially spliced genes in *Riccia fluitans*

**DOI:** 10.3389/fpls.2026.1874689

**Published:** 2026-09-23

**Authors:** Mateusz Maździarz, Katarzyna Krawczyk, Paweł Sulima, Jakub Sawicki

**Affiliations:** 1 Department of Botany and Evolutionary Ecology, University of Warmia and Mazury in Olsztyn, Olsztyn, Poland; 2 Department of Genetics and Plant Pathophysiology, University of Warmia and Mazury in Olsztyn, Olsztyn, Poland

**Keywords:** 5-methylcytosine, adaptation, alternative splicing, *Riccia fluitans*, sequencing

## Abstract

Phenotypic plasticity in amphibious plants was considered a key evolutionary strategy for survival in fluctuating environments; however, the epigenetic mechanisms and post-transcriptional regulations governing the transition between aquatic and terrestrial life forms were previously poorly understood. In the present study, the interplay between 5mC DNA methylation and alternative splicing (AS) was investigated in *Riccia fluitans*, a species exhibiting distinct morphological and transcriptomic adaptations to different habitats. To achieve this, Illumina short-read sequencing was integrated with Oxford Nanopore Technologies direct RNA and direct DNA long-read sequencing. A total of 19,045 AS events were detected, comprising 4,927 A3SS, 3,580 A5SS, 169 MXE, 5,814 RI, and 4,555 SE. Among these, 1,251 events were found to be specific to the water form, 845 events were identified as specific to the land form, and 448 events were determined to differ significantly between the two forms. Furthermore, it was demonstrated by 5mC DNA methylation profiling that methylation conserved in both forms was concentrated in CG motifs, whereas habitat-specific methylation was found to occur primarily in the dynamic CHH context, with higher levels being reached in the water form. A decrease in methylation levels in the flanking regions of genes undergoing shifts in AS was observed, which was interpreted as an indication that environment-dependent reprogramming of 5mC methylation co-occurred with the regulation of AS.

## Introduction

Phenotypic plasticity—the capacity of a single genotype to express distinct, reversible phenotypes across environments—is a core component of plant adaptation to fluctuating water availability (Chinnusamy and Zhu, 2009). Bryophytes are particularly informative for dissecting environment-driven regulatory programs because they represent early-diverging land plant lineages and retain life-history and developmental features that illuminate key steps in terrestrialization (Jonathan Shaw et al., 2011; Shen et al., 2025). The amphibious liverwort *Riccia fluitans* is an especially tractable system in this context: a single genotype can adopt morphologically and anatomically distinct water form and land form thalli, enabling controlled experiments that isolate environmentally induced molecular changes from standing genetic variation (Althoff and Zachgo, 2020; Althoff et al., 2022). In the research on *R. fluitans*, it was demonstrated that this plant adapts to changes in terrestrial and aquatic environments through independent, dynamic processes at the transcriptional, post-transcriptional, and epigenetic levels (Maździarz et al., 2024a, b, 2025, 2026). Wide-ranging changes in RNA expression were included in the key mechanisms of this plasticity, and the precise regulation of transcript stability and translation was achieved through modifications of poly(A) tail length and methylation levels of N6-methyladenosine (m6A), 5-methylcytosine (m5C), and pseudouridine (Ψ) (Maździarz et al., 2024a, b, 2025). Furthermore, 6mA methylation was identified for the first time in the organellar genomes of this plant (with higher levels being observed in the water form), by which a previously unknown, multi-compartmental system of epigenetic regulation in early land plants was revealed (Maździarz et al., 2026). The discovery of these intricate regulatory systems highlights the unique advantage of using *R. fluitans* over traditional systems. Specifically, while *Marchantia polymorpha* was long maintained as the primary model in bryophyte genetics, its utility in exploring the water-to-land transition was limited (Poveda, 2020). In contrast, *R. fluitans* was rendered an unparalleled system for elucidating the mechanisms underlying environmental plasticity by its unique amphibious lifestyle and reversible morphological transformation (Althoff et al., 2022).

A central, still incompletely resolved question is how environmental cues are translated into coordinated transcriptome remodeling in early land plants. Beyond differential gene expression, alternative splicing (AS) provides a pervasive and fast-acting regulatory layer that expands transcript diversity, remodels coding potential, and modulates RNA stability, localization, and translation (Staiger and Brown, 2013; Reddy et al., 2013). In plants, AS is widespread and often dominated by intron retention, with important consequences for open reading frames and nonsense-mediated decay (Ner-Gaon et al., 2004; Reddy et al., 2013). Numerous studies in angiosperms demonstrate that AS is highly responsive to environmental perturbations (e.g., temperature shifts), producing rapid isoform-ratio changes that can precede or occur independently of large gene-level expression changes (Laloum et al., 2018; Calixto et al., 2018). Mechanistically, AS can rewire stress and developmental pathways by altering protein domains, regulatory motifs, subcellular targeting, or by selectively downregulating transcripts through premature termination codons (Staiger and Brown, 2013; Laloum et al., 2018). Under this regulatory framework, although *R. fluitan*s features a lower average intron density (Levins et al., 2025) than *M. polymorpha* (Bowman et al., 2017), it exhibits a marked evolutionary expansion in average intron length. Notably, the average intron size in *R. fluitans* exceeds the structural parameters characteristic of both *M. polymorpha* and the strictly terrestrial *R. sorocarpa* (Krawczyk et al., 2025).

These properties make AS an attractive candidate mechanism for mediating amphibious plasticity in *R. fluitans*. Switching between aquatic and terrestrial growth entails coordinated changes in gas exchange, surface properties, anchorage, and water balance (Althoff et al., 2022). Because AS can remodel functional outputs without requiring new gene content—and can do so reversibly—it provides a plausible molecular “tuning layer” for habitat-dependent phenotypes. Importantly, AS is increasingly recognized as part of an integrated gene-regulatory continuum in which splice-site choice is frequently co-transcriptional and sensitive to chromatin state and RNA polymerase II (Pol II) kinetics (Kornblihtt et al., 2013; Naftelberg et al., 2015). In plants, direct evidence for kinetic coupling has been provided by research showing that light-dependent changes in Pol II elongation can drive nuclear AS responses, linking environmental signaling to splice-site choice via transcription dynamics (Godoy Herz et al., 2019). At the same time, phenotypic plasticity is widely shaped by epigenetic regulation, including cytosine DNA methylation (5mC), which influences chromatin organization, transposon silencing, and gene regulation across plant genomes (Law and Jacobsen, 2010). Plant 5mC occurs in CG, CHG, and CHH sequence contexts, maintained and remodeled by partially distinct enzymatic pathways, enabling both stable propagation and conditional reprogramming of methylation landscapes under changing environments (Law and Jacobsen, 2010). Stress and habitat transitions can be accompanied by methylome changes, positioning DNA methylation as a candidate interface between environmental inputs and transcriptional/post-transcriptional outputs (Chinnusamy and Zhu, 2009). In bryophytes, methylation systems and genome defense strategies can differ from those of flowering plants; for example, *M. polymorpha* exhibits distinctive methylation and transposon-control features, underscoring that “angiosperm-centric” models may not translate directly to early-diverging land plants (Aguilar-Cruz et al., 2019; Bowman et al., 2022). A growing body of evidence indicates that DNA methylation is not only a transcriptional regulator but can also be coupled to splice-site choice and exon definition. Comparative methylome analyses show that exons—particularly regions near splice junctions—often display higher methylation than surrounding introns, and a substantial subset of alternative exons can be methylation-sensitive (Lev Maor et al., 2015). Several non-mutually exclusive mechanisms have been proposed. First, methylation can be “read” by methyl-binding proteins and chromatin-associated factors that recruit or stabilize splicing regulators at specific loci, thereby biasing exon inclusion/exclusion decisions (Lev Maor et al., 2015; Naftelberg et al., 2015). Second, methylation can influence nucleosome occupancy and chromatin accessibility, which in turn affects splice-site recognition and co-transcriptional spliceosome assembly; nucleosome positioning has been shown to associate with distinct AS outcomes in plants (Jabre et al., 2021). Third, methylation-related chromatin changes can modulate Pol II elongation kinetics—a known determinant of alternative exon inclusion—providing a mechanistic bridge between epigenetic marks and kinetic coupling models of AS (Kornblihtt et al., 2013; Godoy Herz et al., 2019). Genome-wide studies further support functional connections between methylation and splicing in plants, including associations between methylation patterns and splice-site behavior in maize and experimentally supported links between methylation perturbation and AS in rice (Regulski et al., 2013; Wang et al., 2016). Technological advances now enable joint interrogation of methylation and splicing at genome scale. Oxford Nanopore sequencing can detect 5mC directly from ionic current signals without bisulfite conversion, supporting genome-wide methylation profiling while preserving long-read context across repetitive and structurally complex regions (Simpson et al., 2017).

In this study, it was hypothesized that 5mC DNA methylation constitutes a regulatory layer that modulates AS in response to environmental change. Specifically, it was predicted that the level and pattern of 5mC within genes undergoing splicing undergo habitat-dependent reprogramming, suggesting an epigenetic involvement in post-transcriptional regulation. To test this, Oxford Nanopore native DNA sequencing was applied to map 5mC profiles within genes exhibiting differential AS, and these patterns were compared between the land and water forms of *R. fluitans*. The primary objectives were to identify habitat-specific AS patterns and to determine which biological processes, with a particular focus on carbohydrate metabolism, were regulated through these mechanisms in each form. *R. fluitans* was presented here as an evolutionary model for studying how early land plants utilized epigenetic and post-transcriptional strategies to colonize diverse environments. This work provided new insights into the interplay between 5mC DNA methylation and splicing, highlighting the role of this mechanism in the environmental adaptation of early embryophytes.

## Materials and methods

### *Riccia* cultivation and experimental design

*R. fluitans* line RFUWM1 (Levins et al., 2025) was maintained as an axenic *in vitro* culture and was propagated under the standardized land–water induction regime established in our earlier work (Maździarz et al., 2024a). Briefly, apical fragments from sterile stock cultures were used as secondary explants and were transferred to fresh ½-strength Gamborg B5 (½GB5) medium supplemented with 20 g·L^−1^ sucrose and solidified with 8 g·L^−1^ agar-agar (pH 6.0). These were arranged as five explants per plate, separated by ~1–2 cm. Cultures were grown in climate chambers at 24 °C under long-day conditions (16 h light/8 h dark) for four weeks to obtain uniform starting biomass. The common-genotype experiment was then initiated by splitting cultures into two environmental treatments: (i) an aquatic treatment, in which plates were overlaid with sterile deionized water to induce the submerged “water form”, and (ii) a terrestrial treatment, which was maintained on the original solid medium to induce the “land form”. The treatment phase was conducted for two weeks with independent biological replicates per condition (n=4 for land, n=4 for water). Subsequently, whole thalli was harvested for downstream short-read RNA-seq–based AS profiling, direct RNA nanopore sequencing (DRS), and nanopore-based 5mC methylation analysis. For short-read RNA-seq and DRS, four water and four land *R. fluitans* were utilized. For native DNA sequencing and 5mC methylation profiling, four water and three land replicates were processed.

### Nucleic acid extraction and library preparation

For each biological replicate, total RNA was isolated using the RNA Plant Mini Spin Kit (Qiagen, Hilden, Germany) and genomic DNA was extracted using the DNeasy Plant Mini Kit (Qiagen), both following the manufacturer’s protocols and consistent with our established *R. fluitans* workflow (Maździarz et al., 2024a, 2025). The quantity and integrity of the nucleic acids were assessed using an Agilent TapeStation system (High Sensitivity RNA and Genomic DNA ScreenTapes) and a Qubit 4 fluorimeter (HS RNA and BR DNA assays) prior to library preparation. Short-read RNA-seq libraries were constructed using the TruSeq RNA workflow with a Ribo-Zero depletion step and were sequenced on an Illumina NovaSeq 6000 platform (Macrogen Inc., Seoul, Korea). To independently validate significant AS events, native RNA (direct RNA) sequencing libraries were prepared from selected RNA samples using the ONT Direct RNA Sequencing Kit (SQK-RNA002), where poly(A)+ mRNA was enriched using the NEBNext Poly(A) mRNA Magnetic Isolation Module and 50 ng was utilized as input for cDNA strand synthesis with SuperScript III Reverse Transcriptase, followed by adapter ligation with T4 DNA ligase and sequencing on a MinION Mk1C using FLO-MIN106 (R9.4.1) flow cells primed with the EXP-FLP002 kit. Simultaneously, long-read DNA libraries were constructed for 5mC profiling using the Native Barcoding Ligation Sequencing Kit (SQK-LSK114) and the NEBNext^®^ Companion Module for ONT Ligation Sequencing (NEB) and were sequenced on a PromethION P2 Solo device using dedicated R10.4.1 flow cells. All resulting data were used for downstream AS profiling and 5mC methylation calling in the land versus water comparison.

### AS analysis

Raw FASTQ files obtained from Illumina sequencing were trimmed to a length of 140 bp using the Trimmomatic tool v.0.39 (Bolger et al., 2014). Subsequently, the processed reads were mapped to the *R. fluitans* reference genome (JBHFFA01) using the STAR aligner v.2.7.11a (Dobin et al., 2012). The resulting BAM files were sorted and indexed with SAMtools v.1.10 (Li et al., 2009). Following the alignment, AS events were identified and quantified using the StringTie v.2.2.1 (Pertea et al., 2015) and rMATS software v.4.1.2 (Shen et al., 2014). AS events were classified into five distinct types: skipped exon (SE), retained intron (RI), alternative 3′ splice site (A3SS), alternative 5′ splice site (A5SS), and mutually exclusive exons (MXE). The detected AS events were subjected to a multi-step filtering and classification procedure. Initially, events with an Inclusion Level (IncLevel) below 0.1 in at least three out of four replicates in both land and water samples were considered low-confidence and were categorized as ‘others’. AS events were designated as ‘land-specific’ if the IncLevel was ≥ 0.1 in at least three out of four land replicates while failing to reach this threshold in the water samples. A reciprocal criterion was applied to define ‘water-specific’ events. Events maintaining an IncLevel above 0.1 in both environments were classified as ‘intersected’. Finally, these intersected events were identified as differentially alternative splicing (DAS) only if the False Discovery Rate (FDR) was < 0.05 and the absolute Inclusion Level Difference (∣IncLevelDifference∣) was determined to be > 0.1. Furthermore, DAS events were categorized based on the direction of the splicing shift: those with an IncLevelDifference > 0.1 were designated as ‘higher’, while events with a IncLevelDifference < −0.1 were classified as ‘lower’. To simplify biological inference, water-specific AS events were merged with DAS events categorized as ‘lower’ to represent the water-representative dataset. Similarly, land-specific AS events and ‘higher’ DAS events were combined to characterize the land-representative dataset. Subsequently, the consequences of AS were investigated using the TransDecoder and CPC2 (Kang et al., 2017) programs. TransDecoder (https://github.com/TransDecoder/TransDecoder) was utilized to determine Nonsense-Mediated Decay (NMD) targets based on the 50-nucleotide rule, whereas CPC2 was employed to evaluate the coding potential of transcripts derived from the land-representative dataset and the water-representative dataset (**Figure 1**).

**Figure 1:**
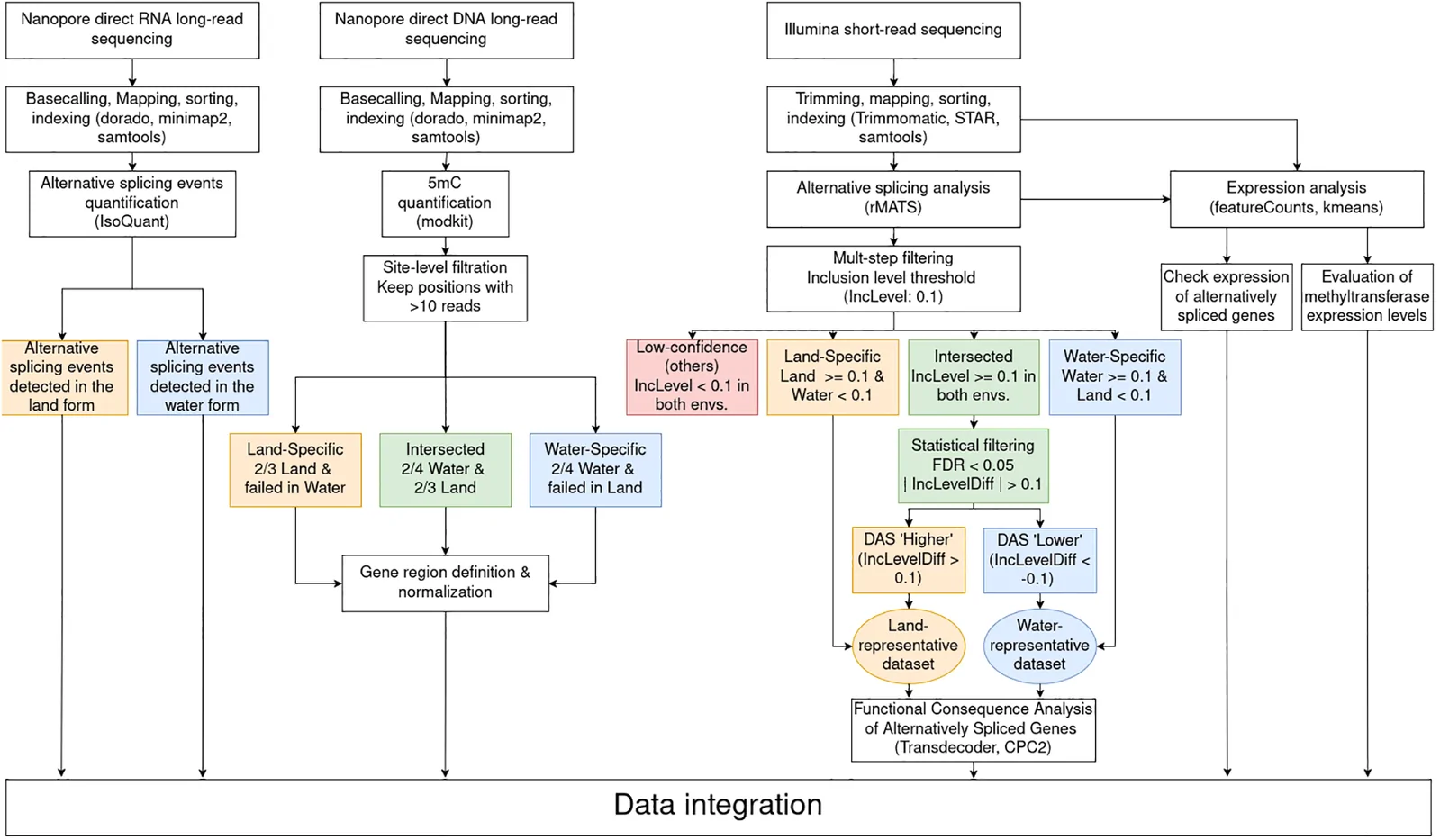
Flowchart illustrating the computational workflow for the integration and analysis of multi-omics sequencing data.

### Association between AS modalities and gene expression strata

The gene expression profile, in the form of raw read counts, was extracted from previously mapped Illumina short-read sequences (BAM files) using the featureCounts v.2.0.6 tool (https://doi.org/10.1093/bioinformatics/btt656). Subsequently, the obtained counts were normalized to Transcripts Per Million (TPM) values separately for the land and water forms of *R. fluitans*. To stratify the genes into three expression groups (low, medium, and high), the k-means clustering algorithm was applied (via the kmeans function from the stats package, v.4.5.2), which was fed with the average TPM values. The decision to employ unsupervised machine learning was dictated by the intention to avoid arbitrary and artificial imposition of expression thresholds, which allowed the algorithm to mathematically determine optimal partitioning boundaries independently for both phenotypic forms. In the next stage, genes in which AS events were identified were assigned to the expression groups determined by the k-means algorithm. For genes exhibiting Intersected AS, the expression profile analysis was performed symmetrically in both environments (based on data from both the land and water forms). In the case of environmentally specific events (land-specific and water-specific), expression levels were analyzed exclusively within the specific ecological form of *R. fluitans* in which the respective splicing event was identified. The expression levels of DNA methyltransferases were also evaluated, including MET1 (methyltransferase 1) and its cofactor VIM1 (variant in methylation 1; CG context), CMT3 (chromomethylase 3; CHG context), CMT2 (chromomethylase 2; CHG and CHH maintenance), and DRM2 (domains rearranged methyltransferase 2; responsible for both *de novo* and maintenance methylation across all three sequence contexts; **Figure 1**).

### Qualitative orthogonal confirmation of splicing architecture using nanopore direct RNA sequencing

To provide independent evidence for the AS events identified by rMATS, Nanopore DRS was employed. A total of eight replicates (four water and four land) were sequenced and subsequently merged into environment-specific pools to maximize transcriptional depth and enhance the detection of full-length isoforms. The raw reads were basecalled with Dorado v.0.4.3 (https://github.com/nanoporetech/dorado) using the rna002_70bps_hac@v3 model. Subsequently, AS events were characterized using IsoQuant v.3.12.2 (Prjibelski et al., 2023), utilizing the GTF annotation previously generated via StringTie from short-read data. The long-read data served as a qualitative reference to confirm the structural consistency of the identified splicing patterns. To ensure a conservative comparison and avoid redundancy, in instances where a single gene contained multiple occurrences of the same AS event type, these were treated as a single record within the supporting dataset (**Figure 1**).

### Gene homology and functional annotation

As *R. fluitans* is a non-model organism, functional annotation was performed by identifying homologs in *Arabidopsis thaliana*. Transcriptome sequences were extracted from the *R. fluitans* genome using the gffread tool v.0.12.7 (Pertea and Pertea, 2020). These sequences were subsequently utilized as queries in a BLASTX v.2.9.0 (Altschul et al., 1990) search against the *A. thaliana* protein database with an e-value threshold of 1×10−5. The BLASTX results were processed to assign a single best *A. thaliana* hit to each *R. fluitans* gene. The resulting *A. thaliana* gene identifiers were then used as input for the gprofiler2 package v.0.2.4 (Kolberg et al., 2023) to conduct Gene Ontology (GO) enrichment analysis. Statistical significance was determined using an adjusted p-value (adj. p-val) threshold of < 0.05. Furthermore, to gain a comprehensive overview of the functional landscape associated with specific splicing mechanisms, genes were categorized according to their respective AS event types. Given the non-model nature of *R. fluitans*, an exploratory screening of the GO and Kyoto Encyclopedia of Genes and Genomes (KEGG) databases was conducted for each AS category independently (Kanehisa et al., 2016). In this specific qualitative assessment, all identified terms were documented regardless of their adjusted p-value significance. This approach was adopted to ensure that biologically relevant trends associated with distinct AS strategies were not overlooked due to the inherent limitations of cross-species functional annotation (**Figure 1**).

### 5mC DNA methylation analysis

Nanopore DNA sequencing POD5 files were basecalled using Dorado v.0.8.1 (dna_r10.4.1_e8.2_400bps_sup@v5.0.0), and sequences were aligned to the *R. fluitans* reference genome using the integrated aligner function. The resulting BAM files were subsequently sorted and indexed using SAMtools. Methylation information, specifically regarding 5mC sites, was extracted from the BAM files utilizing modkit v.0.6.1 (https://github.com/nanoporetech/modkit). To ensure the reliability of the methylation data, sites were separated by strand to prevent the conflation of distinct methylation signals at the same genomic position. Any methylation site that did not align with the reference genome was excluded from the analysis. Furthermore, a stringent filtering process was applied to account for the unequal number of replicates in each group. A methylation site was retained only if it exhibited more than ten methylated reads in at least two out of four water replicates or at least two out of three land replicates. Methylation sites that met these criteria in both environmental conditions were classified as intersected sites. Following this filtration, sites meeting the criteria exclusively in one condition were classified as form-specific. Specifically, sites that passed the threshold in the land-form but failed to meet the criteria in the water-form were designated as land-specific. Conversely, sites that were robustly identified in the water-form but lacked sufficient counterparts in the land-form were assigned as water-specific. To determine the methylation levels within genes undergoing AS (DAS, water-specific, and land-specific), each gene was defined based on its longest transcript variant, including 1,000 bp flanking regions. To standardize varying gene lengths, the gene body and flanking regions were normalized to a relative scale (0–100%). The methylation level was first calculated within each 1% bin as the ratio of methylated cytosines to the total number of cytosines. To obtain the final profile, data were smoothed using a 5-bin rolling mean, representing a sliding window of 5% of the total region length (**Figure 1**).

### Visualization

Sashimi plots were generated using rmats2sashimiplot v.3.0.0 (https://github.com/Xinglab/rmats2sashimiplot) and ggsashimi v.1.1.5 (Garrido-Martín et al., 2018). Circos plots were produced using the circlize library v.0.4.17 (Gu et al., 2014). All remaining visualizations were created with the ggplot2 package v.4.0.2 (Wickham, 2016). KEGG pathways were visualized using the pathview package v.1.50.0 (Luo and Brouwer, 2013).

## Results

### Baseline transcriptional activity and expression patterns of DNA methyltransferases

To characterize the transcriptional landscape of *R. fluitans*, global gene expression levels were first compared between the land form and the water form. A larger pool of actively transcribed genes was identified in the water form (36,109) compared to the land form (26,168), and most genes were assigned to the “Low” expression category in both conditions (**Supplementary Figures 1A; B**, **Supplementary Table 1**). Given the potential role of DNA methylation in phenotypic plasticity, the expression patterns of key DNA methyltransferases (MET1, CMT3, CMT2, and DRM2) and cofactor VIM1 were further evaluated. A general trend of higher or stable expression of these enzymes was observed in the water form (**Supplementary Table 1**). Specifically, while the maintenance machinery (MET1 and VIM1), CMT3 and the *de novo* methyltransferase DRM2 were maintained at stable levels or showed increased expression in the water form, CMT2 (operating as a maintenance methyltransferase in CHG and CHH contexts) was found to be notably absent in the land form. These expression patterns indicated that the transition to the land form was associated with a reconfiguration of the epigenetic machinery, particularly concerning CHG and CHH methylation pathways (**Supplementary Table 1**).

### Comparative analysis of AS landscapes and functional GO enrichment in water and land forms of *R. fluitans*

To contextualize the AS dynamics in *R. fluitans*, the general prevalence of splicing modalities in the kingdom Plantae was first considered. Unlike in Metazoa where SE is represented as the primary AS mechanism, RI was established as the most predominant and evolutionarily conserved event across diverse plant lineages (Petrillo, 2023; Zhou et al., 2024; Du et al., 2024). A total of 19,045 AS events were identified in *R. fluitans*, with the highest frequencies observed for RI, A3SS and SE (**Figures 2A, E**). Based on the established classification, 14,442 events were categorized as “Intersected,” 1,251 as “Water-specific,” 845 as “Land-specific,” and 2,507 were classified as “Other” and excluded from further analysis (**Figure 2B**). Furthermore, a higher frequency of each AS event type was observed in the water form compared to the land form, with A3SS and RI identified as the most prevalent types (**Figure 2C**; **Supplementary Table 2**). AS-containing genes were predominantly assigned to the “Medium” expression class, while a smaller proportion was identified in the “Low” class (**Supplementary Figure 1C**). A total of 1,251 water-specific events were identified in 1,008 genes (861 unique and 147 shared), while 845 land-specific events were located in 724 genes (577 unique and 147 shared; **Figures 2B, D**).

**Figure 2:**
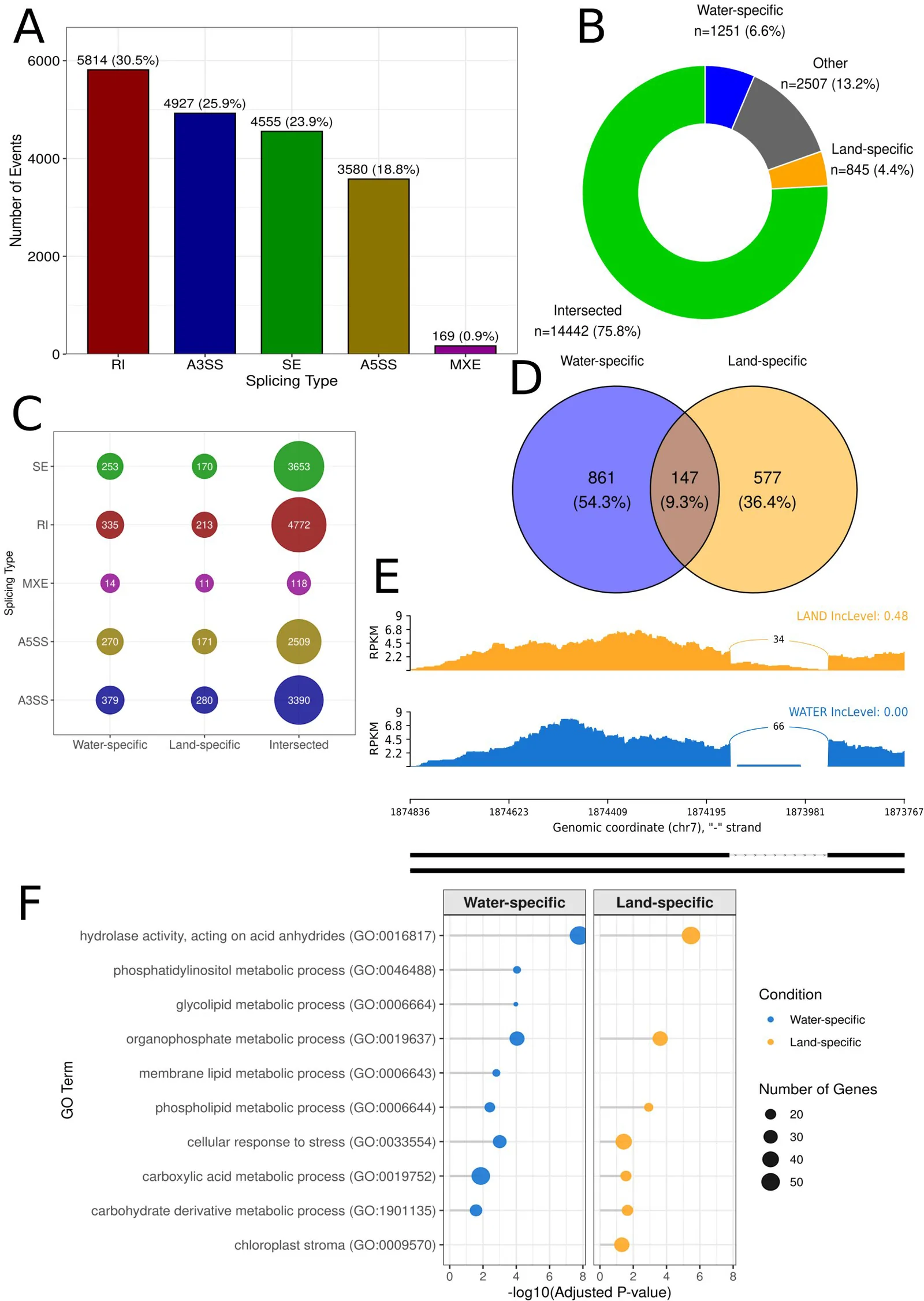
Global and specific AS landscape in *R. fluitans*. **(A)** Barplot was used to visualize the total number of detected splicing events. These were categorized into: Intron Retention (RI, red), Alternative 3’ Splice Site (A3SS, blue), Skipped Exon (SE, green), Alternative 5’ Splice Site (A5SS, yellow), and Mutually Exclusive Exons (MXE, purple). **(B)** The proportional share of AS events was represented by a donut plot. Events were classified into water-specific, land-specific, intersected, and other categories. **(C)** Balloon plot was generated to display the frequency of AS types (y-axis) within the intersected, land-specific, and water-specific groups (x-axis). Dot sizes were scaled according to event counts; colors were assigned based on the types defined in panel **(A, D)** Venn diagram was constructed to show the intersection of genes associated with condition-specific AS events. Water-specific and land-specific genes were highlighted in blue and yellow, respectively. **(E)** Sashimi plot was created for a selected RI event in the CL.27047 gene. Genomic coordinates were plotted on the x-axis, while RPKM values were displayed on the y-axis. Land and water forms samples were colored orange and blue, respectively. **(F)** Lollipop plot was developed to show GO enrichment for condition-specific genes. GO terms were listed on the y-axis, and statistical significance was expressed as −log10 (Adjusted P-value) on the x-axis. Circle sizes were determined by the number of genes associated with each term.

To analyze genes exhibiting specific AS, the homology of *R. fluitans* to *A. thaliana* was evaluated. Out of 74,736 *R. fluitans* transcripts used as queries, significant hits against the *A. thaliana* protein database were produced by 33,147 (44.35%) transcripts within the specified e-value threshold (1×10−5). Distinct functional profiles were observed between the two morphotypes through GO enrichment analysis. In the land form, AS was preferentially directed toward photosynthesis-related structures, as evidenced by an enrichment for chloroplast stroma (GO:0009570). Conversely, in the water form, AS regulation was shifted toward cellular signaling and structural adaptations, particularly those involving membrane lipid remodeling. Despite these divergences, core survival mechanisms, such as hydrolase activity (GO:0016817) and cellular response to stress (GO:0033554), were maintained across both environments (**Figure 2F**; **Supplementary Table 3**). Overall, it was indicated by these findings that while core metabolic and stress responses were maintained across both habitats by AS, this mechanism was dynamically deployed to modulate chloroplast on land versus membrane lipid remodeling and signaling in water, whereby the distinct physiological demands of terrestrial and aquatic lifestyles were reflected.

### RI and A3SS selection drive the transcriptomic divergence between land and water forms of *R. fluitans*

AS events classified as “Intersected” were analyzed to evaluate the statistical significance of differences between the land and water forms of *R. fluitans*. A total of 448 DAS events were identified, of which 244 were categorized as higher inclusion and 204 as lower inclusion. Regarding the distribution of splicing types, RI was identified as the most frequent (192 events), followed by A3SS (106), A5SS (84), and SE (63), whereas MXE was observed as the least common type (3 events) (**Figures 3A, B**). Although RI was established as the most frequent AS event within the DAS, it was revealed by the classification based on IncLevelDifference that higher inclusion levels were exhibited by the majority of these RI events in the land form (**Figures 3A, B**; **Supplementary Table 4**). An inverted trend was indicated within the “Intersected” dataset compared to the AS-specific cohort, by which it was suggested that isoform diversity in the land form was more actively shaped by RI events shared by both forms.

**Figure 3:**
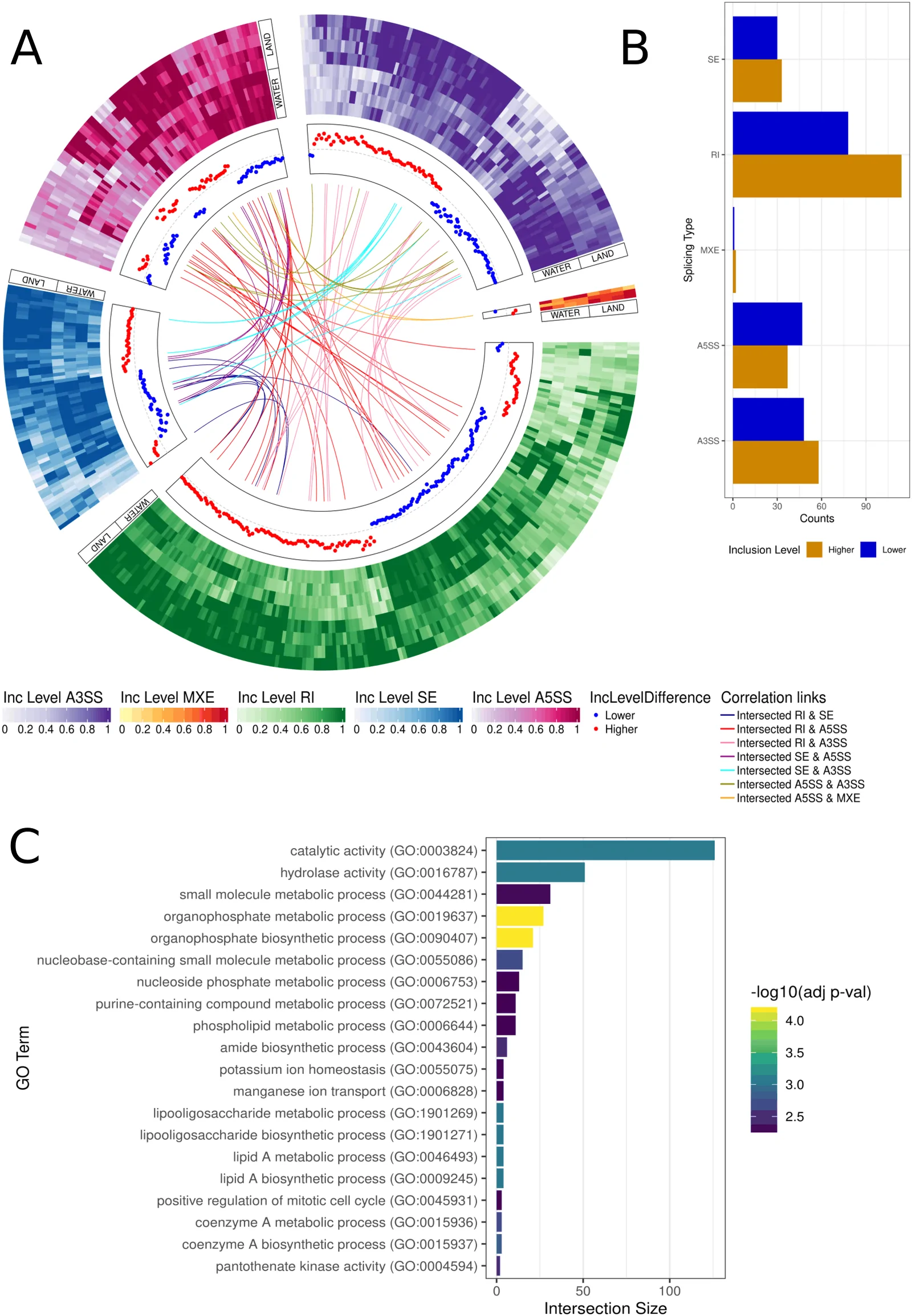
Comprehensive analysis of DAS and functional enrichment. **(A)** A circular heatmap was employed to illustrate the inclusion levels of DAS events. Splicing types were color-coded as follows: green for Intron Retention (RI), blue for Skipped Exon (SE), pink for Alternative 3′ Splice Site (A3SS), purple for Alternative 5′ Splice Site (A5SS), and yellow for Mutually Exclusive Exons (MXE). The second track was utilized to highlight the Inclusion Level Difference between groups, while the innermost track was used to identify common genes associated with multiple splicing event types. **(B)** Distribution of DAS event directions. Bar plots were generated to show the frequency of individual splicing types. The y-axis was used to list AS types (A3SS, A5SS, RI, SE, and MXE), while the x-axis was assigned to the event count. Events were categorized by their inclusion level, where orange and blue were used to represent ‘Higher’ and ‘Lower’ inclusion levels, respectively. **(C)** Functional enrichment of DAS-associated genes. The top 20 most significant GO terms were visualized using a bar plot. GO terms were plotted on the y-axis, and the number of genes associated with each process was displayed on the x-axis. Bar colors were assigned based on −log10 (adjusted p-value) values, according to the scale provided on the right.

To characterize the biological implications of these variations, a functional enrichment analysis was performed on the genes affected by DAS, by which a highly coordinated modulation of enzymatic pathways and structural components was observed. Among biological processes, the highest statistical significance was detected for organophosphate metabolic (GO:0019637) and biosynthetic (GO:0090407) terms. This was mirrored in the molecular function category by an enrichment of catalytic (GO:0003824) and hydrolase (GO:0016787) activities. Furthermore, a regulatory shift toward lipid metabolism was highlighted through the overrepresentation of lipooligosaccharide and lipid A biosynthetic and metabolic processes (GO:1901271, GO:1901269, GO:0009245, GO:0046493). Additionally, physiological regulation was represented by terms such as potassium ion homeostasis (GO:0055075) and the positive regulation of the mitotic cell cycle (GO:0045931) (**Figure 3C**; **Supplementary Table 5**). In summary, it was established through this functional landscape that the transcriptomic divergence between the two environmental morphotypes is driven by DAS via the systematic tailoring of fundamental enzymatic capacities, membrane lipid configurations, and cellular homeostasis.

### Integration of DAS and environment-specific AS events

Water-specific AS events and DAS events classified as lower were merged into a single dataset representing the water form of *R. fluitans*. Conversely, land-specific and higher DAS events were combined to characterize the land form. The water form dataset comprised a total of 1,455 AS events, while the land form dataset contained 1,089 events. Following the integration of these datasets, A3SS and RI were identified as the most prevalent AS types across both environmental forms, with A3SS established as the dominant modality (**Figure 4A**).

**Figure 4:**
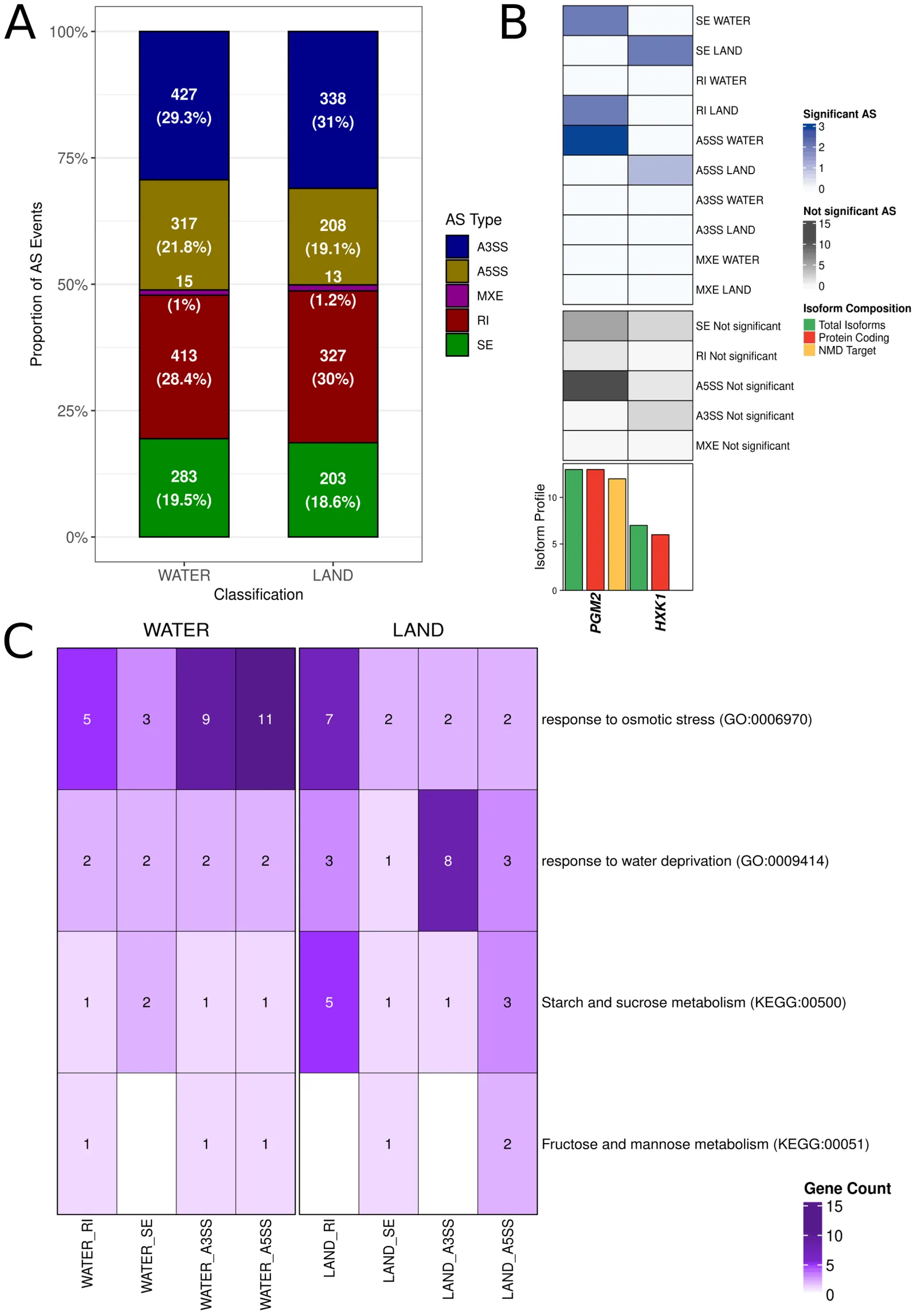
Environment-driven AS patterns and functional profiling in *R. fluitans* water and land forms. **(A)** Stacked barplot illustrating the total number of AS events merged from DAS genes and environment-specific events. The x-axis represents the environment, and the y-axis shows the proportion of each AS event type. AS event types were color-coded as follows: A3SS in blue, A5SS in gold, MXE in purple, RI in red, and SE in green. **(B)** AS event landscape and isoform distribution for PGM2 and HXK1. The heatmaps were used to illustrate the abundance of identified AS events, where color gradients were applied to represent the count of events within each category (a blue scale for significant events and a gray scale for non-significant events). Below, the isoform composition for each gene was depicted by bar plots, where total transcript counts were indicated in green, protein-coding isoforms in red, and NMD-targeted isoforms in yellow. The AS event categories were labeled on the right side of the figure. **(C)** Heatmap of selected GO terms and KEGG pathways enriched for AS events. GO terms/KEGG pathways with their respective IDs are listed on the y-axis, while AS event types divided into RI, SE, A3SS, and A5SS are indicated on the x-axis. Each cell was colored according to the gene count involved in the process, corresponding to the scale bar in the bottom right corner.

To evaluate the self-regulatory capacity of the splicing machinery, AS profiles within transcripts encoding core splicing factors were analyzed. Several key components were identified as being dynamically spliced in a habitat-dependent manner. Most notably, an A3SS event was detected in the RS40 (arginine/serine-rich splicing factor 35, SC35-like, CL.33570, AT4G25500), which was found to be strictly restricted to the water form. Similarly, prominent water form splicing variations were observed in other regulatory transcripts. The AT1G60900 transcript (U2 snRNP auxiliary factor large subunit, CL.23103) was found to undergo a water form MXE event, alongside a land form RI event. Furthermore, a SE event within the AT1G60200 transcript (splicing factor PWI domain-containing protein, CL.18111) was revealed as a water form feature. Complex multi-layered AS patterns were unveiled for the AT2G16940 (Splicing factor CC1-like protein, CL.30979), which was characterized by three distinct, concurrent AS events showing divergent specificities: a water form SE, a land A3SS, and a land SE. Finally, a RI event in the SMP1 transcript (Pre-mRNA splicing Prp18-interacting factor, CL.781, AT1G65660) was classified as a characteristic feature of the water form of *Riccia*. Collectively, an intricate, habitat-driven feedback loop targeting the splicing machinery was suggested by these structural variations during water-to-land transitions (**Supplementary Tables 2**, **4**).

A total of 10,288 isoforms were identified within genes exhibiting water or land form splicing, comprising 8,173 protein-coding and 2,115 non-coding transcripts, among which 6,781 variants were classified as NMD targets, representing an average NMD occupancy of 62.25% per gene across the AS profiles of the water and land forms of *R. fluitans* (**Supplementary Table 6**). A total of 13 isoforms were generated by the PGM2 gene (Phosphoglucomutase/phosphomannomutase family protein; CL.2833), all of which retained an ORF and were classified as protein-coding. However, 12 out of these 13 coding transcripts were concurrently flagged as targets for nonsense-mediated mRNA decay based on the 50-nucleotide rule. Active environmental responsiveness was maintained by PGM2, whereby A5SS (3) and SE (2) were selectively recruited in the water form, while a shift was made exclusively towards significant RI (2) in the land form. In sharp contrast, a highly precise and streamlined splicing architecture was exhibited by the HXK1 gene (hexokinase 1; CL.30286), which was operated entirely without the engagement of the NMD surveillance system. Out of its seven total isoforms, a clean functional bifurcation was featured by HXK1, where six transcripts represented robust, full-length protein-coding variants, while exactly 1 transcript was generated as a strict non-coding RNA. Its environmentally induced differential splicing was kept completely quiescent in the water form but was rendered highly active upon land transition, with significant A5SS (1) and SE (2) being executed. Taken together, it was demonstrated by these results that distinct post-transcriptional modalities were utilized by *R. fluitans*. While a complex AS-NMD feedback loop that paired environmental switching with the production of truncated or buffered coding variants was relied upon by PGM2, a high-fidelity system that cleanly segregated functional coding diversity from non-coding regulatory elements was utilized by HXK1, whereby NMD-mediated degradation was completely avoided (**Figure 4B**; **Supplementary Table 6**; **Supplementary Figure 2**).

Functional enrichment analysis revealed that AS in both morphotypes was associated with osmotic stress and water deprivation responses. This suggests that splicing modulation acts as a conserved regulatory layer for managing the transition between aquatic and terrestrial environments (**Figure 4C**; **Supplementary Table 7**). Diverse AS modalities were observed, as exemplified by genes such as HK1 (histidine kinase 1, CL.891), TEJ (Poly (ADP-ribose) glycohydrolase (PARG), CL.30558), and PGM2 (Phosphoglucomutase/phosphomannomutase family protein, CL.2833), in which a clear shift in splicing patterns between morphotypes was demonstrated. While these genes were regulated via RI in the land form, AS patterns such as A3SS, A5SS, and SE were preferentially recruited in the water form, indicating that the mode of splicing is fine-tuned to the specific environmental context of these genes. Beyond these regulatory genes, distinct AS architectures were observed across central metabolic pathways, particularly starch, sucrose, and fructose-mannose metabolism (KEGG:00500, KEGG:00051) (**Figure 4C**; **Supplementary Figures 3**, **4**; **Supplementary Table 7**). Taken together, it was established by these findings that AS was utilized by *R. fluitans* to rewire fundamental enzymatic capacities, whereby metabolic adaptation to distinct environmental habitats was achieved.

### Distribution and sequence context analysis of 5mC methylation in alternatively spliced genes of *R. fluitans*

The 5mC methylation landscape was characterized within genes that underwent habitat associated AS, based on a total of 386,194 detected methylation positions. These included land specific and higher DAS events, by which the land form was collectively characterized, as well as water specific and lower DAS events, by which the water form of *R. fluitans* was represented. Out of these, 250,557 sites were identified as Intersected, 111,771 were classified as water specific, and 23,866 were determined to be land specific (**Figure 5A**). Across these groups, the CG motif was identified as the dominant context in Intersected sites, whereas distinct prevalence patterns were exhibited by CHG and CHH contexts, which were defined as non CG methylation, within the environment specific subsets (**Figures 5A-D**). Furthermore, a reduced frequency of methylation was observed in the land form compared to the water form. It was suggested by these findings that habitat specific epigenetic regulation in the land form and water form of *R. fluitans* was primarily driven by non CG methylation, whereby more plastic transcriptional responses to aquatic and terrestrial conditions were likely facilitated.

**Figure 5:**
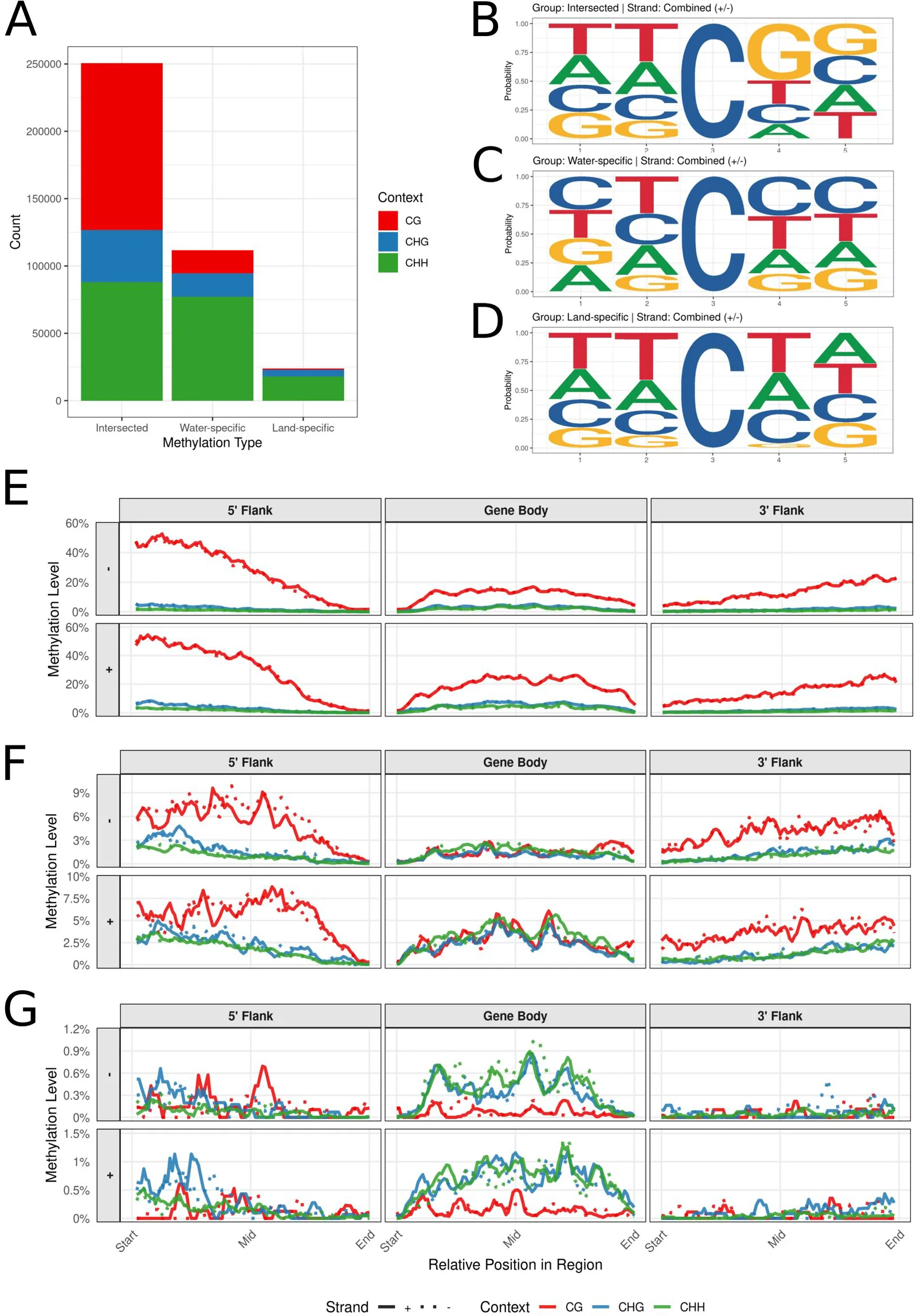
Comprehensive methylation landscape of Intersected, water-specific, and land-specific sites associated with alternatively spliced genes. **(A)** Barplot was generated to represent the total number of methylation sites, ordered from left to right as intersected, water-specific, and land-specific. The methylation type was plotted on the x-axis, while the total count was displayed on the y-axis. The genomic context was color-coded, with red, blue, and green utilized to represent CG, CHG, and CHH contexts, respectively. **(B–D)** Nucleotide sequence motifs (5 nucleotides in length) were analyzed for sites containing methylated cytosines, where the central cytosine was consistently methylated. These motifs were differentiated for both positive (+) and negative (-) strands. **(B)** was assigned to intersected methylation, Panel **(C)** was dedicated to water-specific methylation, was restricted to land-specific methylation sites. (**E-G**). The distribution of cytosine methylation density was analyzed across genes classified as intersected, water-specific, and land-specific. The data were presented in three distinct panels based on methylation subsets: **(E, D)** was utilized to display intersected methylation, **(F)** was focused on water-specific methylation sites, and **(G)** was restricted to land-specific methylation sites. Additionally, the y-axis was vertically partitioned into two sub-panels (+ and -) to represent the genomic strand orientation of the analyzed genes. The genomic context of the cytosines was represented by line colors, with red, blue, and green utilized for CG, CHG, and CHH contexts, respectively. Furthermore, the methylation levels were differentiated by DNA strand orientation, where the positive strand was indicated by solid lines and the negative strand by dotted lines. Each plot was partitioned into three functional segments: the 5’ flanking region, the gene body, and the 3’ flanking region. The relative position within each region, expressed as a percentage, was plotted on the x-axis, while the methylation level was displayed on the y-axis.

The distribution of 5mC within environment-specific and DAS genes was revealed to undergo a decrease in methylation levels in both upstream and downstream flanking regions across intersected, water-specific, and land-specific subsets. In the intersected methylation group, the CG context was found to be dominant within both the gene body and the flanking regions, with the highest methylation levels observed at the beginning of the 5’ flanking region. Similar distribution patterns were recorded for genes located on both the positive and negative strands. Within the gene body, the methylation profile was characterized by an initial increase near the 5’ end, followed by a subsequent decrease toward the 3’ end. In contrast, methylation levels in the CHG and CHH contexts were maintained at consistently low levels across the 5’ flanking region, the gene body, and the 3’ flanking region (**Figure 5E**). The water-specific methylation was found to exhibit a distinct pattern compared to the intersected methylation sites of *R. fluitans*. Within the gene body, the methylation levels were maintained at a relatively constant level across its entire length. In the 5’ and 3’ flanking regions, the Water-specific CG methylation did not show such a prominent divergence as was observed in the Intersected methylation group. Furthermore, the disparities between CHG/CHH and CG methylation levels were markedly reduced in the Water-specific subset compared to Intersected categories (**Figure 5F**). The methylation identified as specific to the land form of *R. fluitans* was found to exhibit a distinct pattern, differing from both the intersected and water-specific groups. Notably, within the gene body, CHG and CHH methylation levels were observed to dominate over the CG methylation level. Furthermore, the 5’ and 3’ flanking regions in this subset were characterized by the lowest methylation levels among all analyzed groups, with all three genomic contexts remaining near 0%, showing no clear dominance of any single context (**Figure 5G**). Collectively, these distinct distribution profiles suggested that unique epigenetic landscapes were established for each morphotype. While the intersected methylation sites were characterized by a stable CG-dominated pattern, the environment-specific subsets displayed specialized methylation architectures, further indicating that the land form and water form of *R. fluitans* were regulated by divergent, habitat-specific epigenetic mechanisms.

To evaluate the epigenetic landscape of core splicing factors undergoing habitat-dependent AS, methylation patterns were analyzed. The splicing factor RS40 (CL.33570, arginine/serine-rich splicing factor 35) was found to be devoid of methylation, and no land-specific methylation was detected on the positive strand in any of the analyzed splicing factors. Detailed analysis revealed that conserved, Intersected methylation sites were strictly confined to the CG context. In contrast, habitat-specific profiles displayed higher non-CG context diversification. Notably, the PWI domain-containing protein (CL.18111, RNA recognition motif (RRM)-containing protein) and the U2 snRNP auxiliary factor (CL.23103, large subunit) exhibited the most diversified water-specific methylation patterns, particularly within their gene bodies and flanking regions. Conversely, limited or strictly Intersected CG methylation was noted for transcripts such as CC1-like protein (CL.30979) and SMP1 (CL.781), with no habitat-specific modifications detected within their gene body regions (**Supplementary Table 8**).

### Consistency and technical limitations in transcriptome reconstruction

Transcript isoforms, poly(A) tail lengths, RNA structures, and abundance were simultaneously captured by DRS in a single experiment, by which a comprehensive view of the transcriptome was offered (Parker et al., 2020; Begik et al., 2021; Kim et al., 2025). Structural verification using the DRS and IsoQuant pipeline identified 156 consistent AS events for the water form and 58 events for the land form (**Figures 6A, B**). Specifically, in the water *R. fluitans* dataset, these confirmed events comprised 2 A3SS, 121 A5SS, and 33 RI (**Figure 6A**). For the land form, the supported splicing architecture included 3 A3SS, 40 A5SS, and 15 RI (**Figure 6B**). The observed overlap between the splicing platforms was primarily constrained by the sequencing depth of the DRS and the occurrence of premature transcript terminations (**Figure 6C**). Consequently, while limited validation was offered by the DRS, the full complexity of certain transcript isoforms could not be exhaustively mapped solely by DRS sequencing due to these technical limitations (**Figure 6C**).

**Figure 6:**
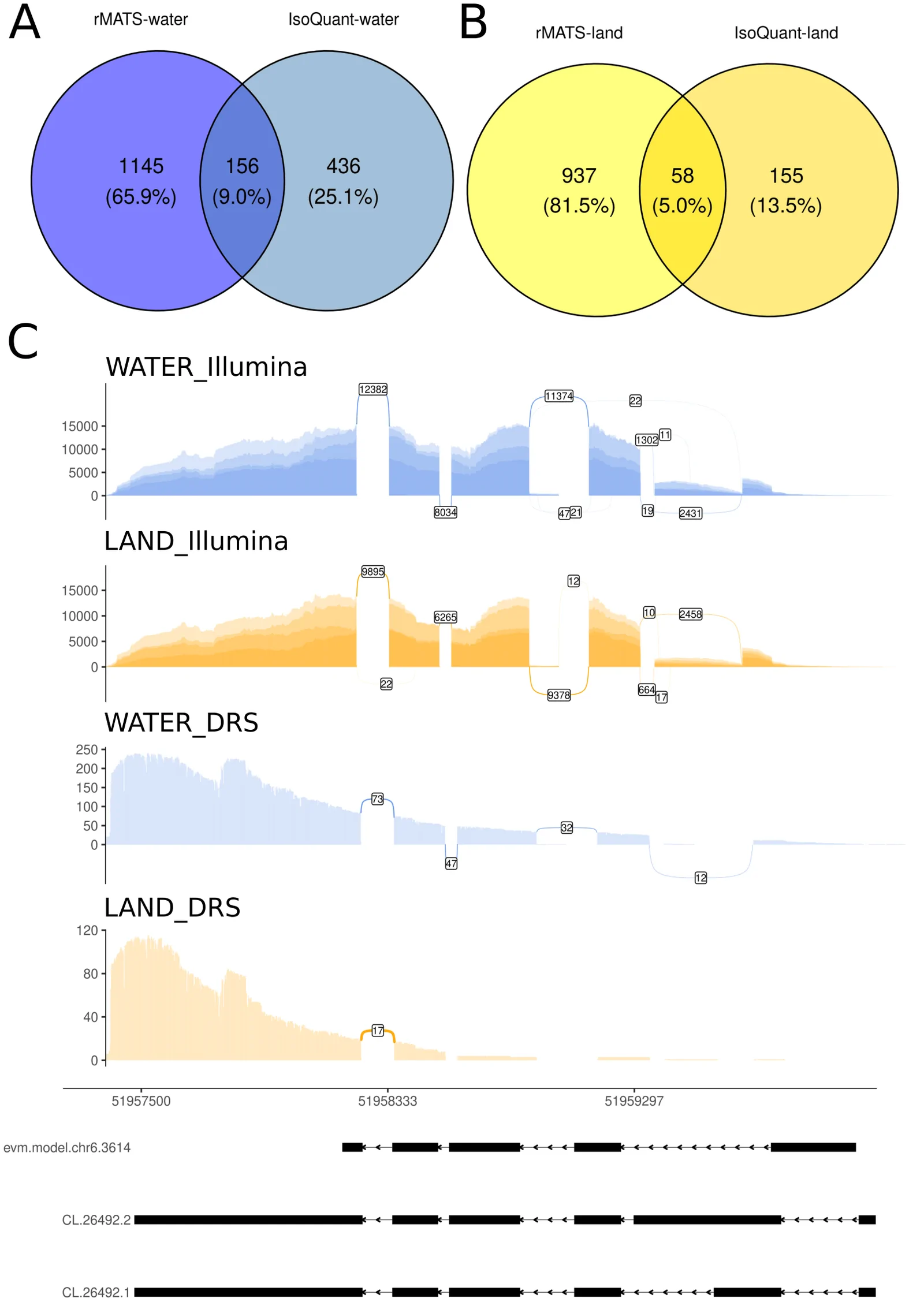
Comparison of AS detection between short-read and direct RNA long-read sequencing technologies in *R. fluitans*. **(A, B)** Venn diagrams displaying the overlap of genes containing identical splicing events (land-specific, water-specific and DAS) identified through short-read technology using the rMATS tool and long-read technology using the IsoQuant tool, for the water form of *R. fluitans* **(A)** and the land form of *R. fluitans* **(B)**. **(C)** Sashimi plot comparing Illumina and DRS sequencing coverage for the gene CL.26492 (AT1G54290, translation initiation factor SUI1 family protein). Values on the arcs represent the number of junction-spanning reads, while histograms illustrate the read coverage depth across exon and intron regions.

## Discussion

In this study, AS was compared between the water and land forms of *R. fluitans*, and the potential influence of 5mC methylation on AS was investigated. The results demonstrated distinct characteristics of AS in both forms of *R. fluitans*. Furthermore, environment-specific methylation patterns, as well as those shared between both forms, were identified within genes exhibiting DAS, land-specific and water-specific AS. Previous investigations of the *R. fluitans* transcriptome revealed a higher density of Ψ, m6A, m5C RNA modifications, alongside a systemic elongation of poly(A) tails in nuclear-encoded transcripts within the water form (Maździarz et al., 2024a, b, 2025). This trend was further corroborated at the organellar level, where elevated levels of 6mA DNA methylation were detected in both plastomes and mitogenomes of the water *R. fluitans* form (Maździarz et al., 2026).

Higher levels of simple sugars, such as glucose, fructose, and maltose, were accumulated by the land form of *R. fluitans*, which likely reflected starch degradation and osmoprotective responses to water deficit, whereas higher levels of sucrose and trehalose were maintained by the water form (Maździarz et al., 2025). Similar metabolic tendencies were exhibited by other plants subjected to flooding or salinity stress (Ram et al., 2002; Yang et al., 2013; Han et al., 2024). These shifts in sugar composition are closely linked to the regulation of key enzymes in central metabolism, including PGM2 and HXK1. In photosynthetic tissues, PGM genes were involved in the conversion of Glc-6-P into Glc-1-P for the production of nucleotide sugars (Sheu and Frey, 1978; Kotake et al., 2004). The role of these genes in carbohydrate distribution was further evidenced by mutations that impaired glycolysis and cell wall biosynthesis (Egli et al., 2010). While the inhibition of cytosolic PGM activity was found to suppress growth in potato (Fernie et al., 2002), it had no effect on *A. thaliana* (Egli et al., 2010), suggesting species-specific metabolic flexibility. In *R. fluitans*, the PGM2 gene, which exhibited RI in the land form and SE as well as A5SS in the water form, was found to produce distinct transcripts (**Figure 4B**; **Supplementary Tables 2**, **4**, **6**) that were differentially activated depending on the environment. Similarly, the HXK1 gene, which is involved in both fructose and mannose metabolism (KEGG:00051) and starch and sucrose metabolism (KEGG:00500), was found to undergo AS (A5SS and SE) exclusively in the land form of *R. fluitans* (**Figure 4B**; **Supplementary Tables 2**, **4**, **6**; **Supplementary Figure 2B**). HXK1 acts as a bifunctional enzyme that serves as both a catalytic agent for glycolysis and a glucose sensor for energy availability (Cho et al., 2006; Wu et al., 2023). In strawberry, higher HXK1 expression was associated with high-sugar varieties where sucrose dominates (Lee et al., 2018; Wu et al., 2023). Consequently, it is suggested that the splicing of HXK1 and PGM2 in *R. fluitans* may modulate kinase activities to facilitate the accumulation of simple sugars or adjust to fluctuating humidity, while a standard metabolic mode is maintained in the aquatic environment.

A relationship between epigenetic structure and the plant’s habitat was revealed through an analysis of 5mC DNA methylation within genes undergoing AS in *R. fluitans*. It was observed that intersected methylation was concentrated mainly in CG motifs, whereas environment-specific methylation was found in CHH motifs (**Figures 5A-D**), with these changes occurring predominantly in the CHH context. Furthermore, it was demonstrated that water-specific methylation was characterized by a higher level than land-specific methylation (**Figure 5A**). This pattern was found to align with the observed expression stability of the DNA methylation machinery in the water form of *R. fluitans*, which was further supported by the notable suppression of the CHH-methyltransferase CMT2 upon transition to the land form (**Supplementary Table 1**). The presence of gene-body CHH methylation in the land form, despite the absence of CMT2 expression, appears contradictory at first, but these findings can be reconciled through alternative non-CG methylation pathways. Specifically, DRM2 remains active in the land form and can mediate non-CG methylation in both CHG and CHH contexts via the canonical RNA-directed DNA methylation (RdDM) pathway (Zhang et al., 2018). This result correlates with reports regarding *Camellia sinensis* L., where CHH-type methylation was shown to be the most dynamic and crucial for abiotic stress response (Kong et al., 2023). The dynamic nature of CHH motifs in the context of developmental regulation and stress response was also confirmed by other studies (Garg et al., 2015; Chiusano et al., 2021), and in the case of flax, it was shown that the number of methylations at CHG and CHH sites increased under the influence of repeated drought stress (Wang et al., 2023). An important observation was made in the present study regarding a decrease in methylation levels in regions upstream of genes exhibiting AS, which differentiated the water from the land form one (**Figures 5E-G**). Although no direct correlation between methylation and DAS genes was detected in tomato during phosphorus deficiency (Tian et al., 2021), our results suggest that this relationship in *R. fluitans* may be indirect. As was demonstrated in wheat, stress factors can influence splicing not only directly, but also through the methylation of genes encoding the splicing factors themselves (Majláth et al., 2025). In our study, fluctuations in 5mC were discovered within the splicing factor genes exhibiting AS (**Supplementary Table 8**). It can therefore be assumed that the observed 5mC methylation co-occurred with changes in land-specific, water-specific AS and DAS. The hypothesis regarding the link between epigenetics and AS processes is supported by studies on *A. thaliana*, where stochastic DNA methylation and nucleosome occupancy levels were linked to distinct splicing profiles in response to cold stress (Jabre et al., 2022). Additionally, a link between AS and the hypermethylation of ONSEN transposon insertions was indicated in *A. thaliana* (Merkulov et al., 2025), suggesting a potential role for transposable elements in shaping the plant transcriptome.

## Study limitations

While this study provided novel insights into the AS and 5mC profiling of *R. fluitans*, certain limitations were identified. The sample size, although in line with comparable exploratory transcriptomic and epigenetic studies, was recognized as a factor that might have limited the statistical power of the analyses. In addition, sampling was restricted to a single time-point per subject, which constrained the temporal interpretation of AS and 5mC shifts. Furthermore, the 5mC profiling was specifically limited to land-specific, water-specific, and DAS genes so that the direct impact of 5mC on AS differences between the land and water forms could be investigated. It was concluded that to fully understand the influence of this specific methylation on AS, the land and water forms of *R. fluitans* should be examined independently as a standalone AS phenomenon within each form. This objective was designated as a focus for future studies. Additionally, the full potential of the native Nanopore DNA sequencing data was not fully exploited, as other DNA modifications such as 6mA, 4mC, and 5hmC were not included in the current analysis. It was acknowledged that these modifications might provide further insights into the regulation of AS differences and will be investigated in subsequent research. Finally, DRS was utilized as a qualitative verification of the mechanism rather than a definitive validation of AS. It was determined that high-depth Nanopore cDNA sequencing should be implemented in future research to rigorously validate the AS sites that were identified via short-read sequencing.

## Conclusions

In this study, a comprehensive insight into the differences in alternative gene splicing between the water and land forms of *R. fluitans* was provided. Specifically, 5mC methylation profiles within these alternatively spliced genes were characterized. The data indicated that unique splicing events and 5mC methylation sites were established in *R. fluitans* in a habitat-dependent manner. Consequently, *R. fluitans* was proposed as a pivotal model organism for studying the evolution of multi-compartmental methylation systems in early land plants.

It was established that *R. fluitans* exhibit a highly plastic transcriptome, with a greater diversity of AS events being shown by the water form than the land form. Distinct metabolic pathways were found to be targeted by habitat-specific splicing, where lipid metabolism was prioritized in water and carbohydrate and chloroplast-related processes were targeted on land, suggesting that AS may represent a pivotal mechanism for environmental adaptation in this species. Furthermore, RI was identified as the primary mechanism by which isoforms are differentiated in response to environmental conditions within DAS events. The metabolic pathways of starch, sucrose, fructose, and mannose were observed to be controlled differently by DAS, land-specific and water-specific genes. A higher abundance of 5mC DNA methylation was characterized in the water form within DAS, land-specific and water-specific genes. Finally, 5mC methylation analysis within DAS, land-specific and water-specific genes revealed that intersected methylations occurred mainly in CG motifs, whereas environment-specific methylations were localized predominantly within CHH motifs.

## Data Availability

The datasets presented in this study can be found in online repositories. The names of the repository/repositories and accession number(s) can be found in the article/**Supplementary Material**.

## Supplementary material

Supplementary Figure 1 Gene expression analysis in the land and water forms of *Riccia fluitans*. **(A, B)** Scatter plots illustrating land expression (TPM) on the x-axis and water expression (TPM) on the y-axis. The data were partitioned according to the expression profiles of the *R. fluitans* water form **(A)** and land form **(B)**. Data points were color-coded according to the legend provided at the bottom of the panel. **(C)** Bar plots presenting the distribution of all AS events, with the types of AS events displayed on the x-axis and their counts on the y-axis, categorized across genes into low, medium, and high expression groups.

Supplementary Figure 2 Sashimi plots of **(A)** PGM2 (CL.2833) and **(B)** HXK1 (CL.30286). Gene expression levels in the water form were highlighted in blue, whereas expression levels in the land form were indicated in orange. Intron and exon structures were delineated at the bottom of each panel.

Supplementary Figure 3 Starch and sucrose metabolism pathway (KEGG:00500) visualized using Pathview library. Boxes represent genes encoding specific enzymes within the pathway. Genes in which splicing events were categorized into the *R. fluitans* land form were highlighted in orange, whereas those assigned to the water form were indicated in blue. Splicing events identified in both the land and water forms were color-coded in green.

Supplementary Figure 4 The Pathview-generated layout of the fructose and mannose metabolism pathway (KEGG:00051). Individual enzymes within the network are denoted by rectangular nodes mapping the corresponding host genes. Elements associated with splicing modifications specific to the land *R. fluitans* are marked in orange, while those exhibiting shifts in the water variant are shaded in blue.
